# Effectiveness of Vitamin D Supplements among Patients Hospitalized for COVID-19: Results from a Monocentric Matched-Cohort Study

**DOI:** 10.3390/healthcare10050956

**Published:** 2022-05-22

**Authors:** Vito Fiore, Andrea De Vito, Paola Bagella, Elija Princic, Anna Antonella Mariani, Lucia Denti, Alessandro Giuseppe Fois, Giordano Madeddu, Sergio Babudieri, Ivana Maida

**Affiliations:** 1 Infectious and Tropical Diseases Clinic, Department of Medical, Surgical and Experimental Sciences, University of Sassari, 07100 Sassari, Italy; andreadevitoaho@gmail.com (A.D.V.); paola.bagella@tiscali.it (P.B.); princic_e@hotmail.com (E.P.); annaanto82@gmail.com (A.A.M.); dentilucia@outlook.it (L.D.); giordano@uniss.it (G.M.); babuder@uniss.it (S.B.); imaida@uniss.it (I.M.); 2 Unit of Respiratory Diseases, University Hospital Sassari (AOU), 07100 Sassari, Italy; agfois@uniss.it

**Keywords:** COVID-19, vitamin D3 supplement, SARS-CoV-2, matched cohort

## Abstract

Objectives: Our study aimed to evaluate the usefulness of Vitamin D3 (VitD3) among patients hospitalized for COVID-19. The primary endpoint was to evaluate the difference in survival rates between patients receiving and not VitD3. The secondary endpoints were to evaluate clinical outcomes, such as needing non-invasive ventilation (NIV), ICU transfer, and laboratory findings (inflammatory parameters). Methods: We conducted a retrospective, monocentric matched-cohort study, including patients attending our ward for COVID-19. Patients were divided into two groups depending on VitD3 administration (Group A) or not (Group B) among patients with low VitD levels (defined as blood levels < 30 ng/mL), which depended on physicians’ judgment. Our internal protocol provides VitD3 100,000 UI/daily for two days. Findings: 58 patients were included in Group A, and 58 in Group B. Patients were matched for age, sex, comorbidities, COVID-19-related symptoms, PaO2/FiO2 ratio, blood exams, and medical treatments. Regarding the principal endpoint, there was a statistically significant difference between the two groups in survival rates [Group A vs. Group B = 3 vs. 11 (*p* = 0.042)]. When considering secondary endpoints, Group A patients were less likely to undergo NIV [Group A vs. Group B = 12 vs. 23 (*p* = 0.026)] and showed an improvement in almost all inflammatory parameters. Conclusions: The link between VitD3 deficiency and the clinical course of COVID-19 during hospitalization suggests that VitD3 level is a useful prognostic marker. Considering the safety of supplementation and the low cost, VitD3 replacement should be considered among SARS-CoV-2 infected patients needing hospitalization.

## 1. Introduction

At the end of December 2019, a new severe respiratory syndrome was described in Wuhan, China [1]. One month later, a new Coronavirus was detected and defined as SARS-CoV-2 [2]. In March 2020, Coronavirus Disease 2019 (COVID-19) was declared a public health emergency by the World Health Organization (WHO) [3]. SARS-CoV-2 enters into the host cells using the angiotensin-converting enzyme 2 (ACE-2) receptor and starts its replication [4,5,6]. After the incubation, a wide spectrum of symptoms could appear; the most common symptoms are fever, cough, and dyspnea, while less common symptoms are fatigue, headache, anosmia, ageusia, cutaneous manifestation, and gastrointestinal symptoms [7,8]. A high risk of disseminated intravascular coagulation and venous thromboembolism, pancreatitis, and depression have also been described [9,10,11,12,13]. After symptom onset, the disease can develop life-threatening systemic inflammation, acute respiratory distress syndrome (ARDS), and multiorgan dysfunction.

Several variables have been associated with worse outcomes among people with COVID-19, in particular, age > 65, pre-existing concurrent cardiological and cerebrovascular disease, D-dimer > 0.5 μg/mL, and ferritin levels [14,15,16,17,18].

Since the start of the pandemic, several treatments have been proposed for COVID-19 (steroids, heparin, remdesivir, monoclonal antibodies, tocilizumab) [19] and to prevent the severe form of the disease (antivirals, monoclonal antibodies, vitamin supplementation) [20]. Many studies focused their attention on vitamin D3 (VitD3) therapeutical use.

As it is well known, VitD3 has both antimicrobial and anti-inflammatory effects. Furthermore, VitD3 increases ACE2 pulmonary expression in acute lung injury animal models [21]. On the one hand, this can increase the cellular viral entry in the first infection phase. On the other hand, this may give potential benefits because of the SARS-CoV-2-mediated downregulation of ACE2 when lung injury is ongoing [22].

Our aim is to describe our experience of VitD3 use among patients hospitalized for COVID-19 in terms of clinical outcomes and survival rates.

## 2. Methods

We conducted a retrospective, monocentric matched-cohort study, including patients attending our ward for COVID-19. Demographical, clinical features, and laboratory findings were collected from patients’ medical records. Patients were divided into two groups depending on VitD3 [25(OH)D3] administration (Group A) or not (Group B) among patients with deficiency or insufficiency (defined as blood levels <30 ng/mL), which depended on physicians’ judgment. Our internal protocol provides VitD3 100,000 UI/daily for two days.

All patients practiced heparin and steroids. In addition, Remdesivir was administered according to national and international indications [23].

### 2.1. Inclusion and Exclusion Criteria

Inclusion depended on adult age (≥18 years), need of oxygen supplement but not in NIV, VitD dosage at admission, inflammatory markers [ferritin (measure: ng/mL), PCR (measure: mg/dL), LDH (measure: mU/mL), and D-dimer (measure: ng/mL)] at admission (T0) and after one week (T1).

Exclusion criteria were age (<18), chronic kidney disease, being in treatment with VitD3 due to other comorbidities, not needing oxygen at admission, and not being able to sign informed consent.

### 2.2. Study Endpoints

The primary endpoint was to evaluate the difference in survival rates between the two groups. The secondary endpoints were to evaluate clinical outcomes regarding non-invasive ventilation (NIV) needing, transfer to ICU, and inflammatory parameters.

### 2.3. Statistical Analysis

Based on a proportional survival difference of 25.5% (92.2% vs. 66.7) [24], statistical power of 80%, an alpha error of 5%, and a 1:1 allocation ratio, a total sample size of 76 patients (38 per group) was needed.

Data distribution was evaluated with the Kolmogorov–Smirnov test before analysis. Data were elaborated as numbers on total (percentages), means ± standard deviations, and median (IQR), as appropriate. Categorical variables were evaluated with the Chi-squared test or Fischers’ exact test as appropriate. Continuous variables were evaluated with the Student’s *t*-test or Mann–Whitney U test based on their parametric or non-parametric distribution.

Statistical significance was based on a two-tailed *p*-value < 0.05. Univariate logistic regression was carried out to evaluate the relationship between demographics, clinical features, laboratory findings, and treatments with survival rates. Variables with *p*-values < 0.15 were included in multivariate analysis.

Statistical computations were carried out with the statistical software STATA version 16.1 (StatsCorp, TX, USA).

### 2.4. Ethical Issues

The study was conducted in accordance with the declaration of Helsinki. Data collection was part of the protocol ‘COVID-19-SS’ n. PG/2020/9411, approved by the Local Ethical Committee, University Hospital of Cagliari. Patients’ data were fully anonymized. All patients signed informed consent.

## 3. Results

### 3.1. Patients’ Demographics and Clinical Features

Overall, 126 patients were included in the study; 58 patients were included in Group A and practiced VitD3, 33 (56.9%) were male, and the mean age was 62.5 + 14.7 years. The most common comorbidities were hypertension (33; 56.9%) and diabetes (16; 27.6%). The symptoms were dyspnea (34; 58.6%), fever (31; 53.4%), and asthenia (28; 48.3%).

Group B (n = 58) patients were matched for sex (male sex = 33; 56.9%) and had a mean age of 62.9 + 12.8 years. The most common comorbidities were hypertension (29; 50%) and diabetes (11; 18.9%), which were the same in Group A. Additionally, in Group B, the most frequent comorbidities were hypertension (32; 55.1%) and diabetes (11; 18.9%), as well as symptoms such as dyspnea (38; 65.5%), fever (32; 55.2%), and asthenia (25; 43.1%).

Regarding inflammatory markers, Group A and B matched on ferritin, D-dimer, and C-reactive protein (CRP). Only lactic dehydrogenase (LDH) significantly differed between the two groups (*p* = 0.03) at T0.

Regarding treatments, all patients in both groups received heparin and steroids (dexamethasone 6 mg). Remdesivir was administered to 40 (68.9%) patients in Group A, and 38 (65.5%) patients in Group B (*p* = 0.69)

The comparison of baseline characteristics between the two groups is reported in Table 1.

### 3.2. Endpoints’ Evaluation

When comparing the two groups’ survival rates, there was a statistically significant difference [Group A vs. Group B = 3 vs. 11 (*p* = 0.042)]. Secondary endpoints showed Group A patients were less likely to undergo NIV [Group A vs. Group B = 12 vs. 23 (*p* = 0.026)], but there were no differences in terms of ICU transfer and invasive ventilation [Group A vs. Group B = 4 vs. 8 (*p* = 0.36)]. Furthermore, Group A showed an improvement in almost all inflammatory parameters. Only ferritin showed a significant increase in Group A (*p* = 0.0058). No significant reduction was observed in Group B.

The inflammatory markers’ comparison between T0 and T1 is reported in Table 2.

At the logistic regression, COPD (*p* = 0.005) and CRP levels (*p* = 0.009) had a positive relationship with mortality. Instead, higher values of the P/F ratio (*p* = 0.033) and VitD3 administration (*p* = 0.015) were related to higher survival rates. Logistic regression analyses results are reported in Table 3.

## 4. Discussion

As previously reported in the literature, patients with low VitD levels have a high risk of ARDS [25]. Additionally, pre-infection levels seem to be predictive of patients’ outcome. In a recent retrospective study, Dror et al. showed how a pre-infection deficiency of VitD was associated with severe illness and patients’ mortality [26]. As a consequence, VitD levels may have an important value in patients’ assessment by physicians.

The VitD immune modulation propriety has been widely studied. VitD synthesis in monocytes and macrophages stimulates cathelicidin expression. Cathelicidin is related to antimicrobial activity [27]. Another VitD-induced activity is related to β-Defensin 2 production. This molecule stimulates both chemokines and cytokines production mediating the hosts’ response to pathogens [28,29]. The cited mechanisms are also involved in viral infections. In fact, both cathelicidin and β-Defensin 2 activity are observed during the hosts’ response to viruses [30].

However, till 2021, the available studies concluded that more data were needed on the real clinical effects of VitD use and benefits in COVID-19 patients [31].

Our study showed a significant protective effect of VitD3 administration in bolus doses among patients hospitalized for COVID-19.

These results demonstrated that in the moderate-severe COVID-19 disease needing hospitalization, oral supplementation of cholecalciferol using 100,000 IU for two days improves both the survival rates and reduces the risk of severe respiratory distress as mirrored by the need for NIV and inflammation markers, compared with patients who did not receive it.

We noticed an increased level of LDH in both groups, and it was higher in Group B. A recent meta-analysis showed how higher LDH levels in COVID-19 patients could be related to worse outcomes [32]. However, in the multivariate analysis, LDH was not associated with an increased risk of death (OR 0.99, CI95% 0.99–1.01, *p* = 0.702).

No association with ICU admission was observed comparing the two groups.

SARS-CoV-2 principally affects the respiratory tract, and data supporting a significant effect of VitD3 in improving respiratory tract infections are well known. Martineau et al. published a meta-analysis in 2017 analyzing 25 randomized studies on the protective effect of VitD3 supplementation among patients with respiratory tract infections [33]. Their results showed how the VitD3 effect was both statistically and clinically relevant.

The immunomodulatory activity of VitD3 has been widely studied. In cases of VitD3 deficiency, immune regulation seems to be less efficient [34,35].

In addition, previous studies suggested that VitD3 replacement could prevent the risk of respiratory failure in patients with COVID-19 [36,37,38]. In this way, our results are concordant with the available literature.

## 5. Conclusions

The link between VitD3 deficiency and the clinical course of COVID-19 during hospitalization suggests that VitD3 level is a useful prognostic marker in addition to the other well-known markers. Considering the safety of supplementation and the low cost, VitD3 replacement should be considered among SARS-CoV-2-infected patients needing hospitalization. VitD3 supplementation could improve patients’ clinical outcomes, as well as reduce hyperinflammation. This study confirms previous data on faster inpatients’ healing after high-dose vitamin D supplementation [39].

## 6. Limitations of the Study

Our study has some limitations. First of all, this is a retrospective study and the time between the onset of symptoms and the administration of vitamin D was not analyzed, and only the time of admission was considered. The count of leucocytes, and especially the neutrophil/lymphocyte ratio, has been described as a marker for infections’ severity. This parameter seems to increase in COVID-19. Given the study was principally focused on clinical outcomes, we did not evaluate NLR. Which could be object of future investigations to evaluate the immunomodulatory effects of VitD after supplementation. Furthermore, VitD blood levels were not checked again after the administration. However, one group underwent supplementation, and one did not receive it. In addition, it is known that spontaneous variation on VitD levels is seasonal. As a consequence, finding a difference in T1 between the two groups, especially when analyzing an acute disease, would represent a bias for the study. Finally, the sample size is relatively small, and further studies are needed to confirm our results, including randomized clinical trials with large sample sizes.

## 7. Strength of the Study

To our knowledge, this is the first matched-cohort study evaluating outcomes among hospitalized COVID-19 patients after VitD3 administration. Furthermore, matching included a broad range of characteristics (demographics, clinical features, laboratory findings, and treatments). As a consequence, even with a small sample, we were able to analyze two strictly matched cohorts, reducing possible biases in the study.

## Figures and Tables

**Table 1:** Baseline characteristics among patients included in our matched-cohort study.

Variable	Group A (*n* = 58)	Group B (*n* = 58)	*p*-Value
Age, mean ± SD	62.5 ± 14.8	62.9 ± 12.8	0.87 *
Male sex, *n* (%)	33 (56.9)	33 (56.9)	1
Diabetes, *n* (%)	16 (27.6)	11 (18.9)	0.27 **
Hypertension, *n* (%)	33 (56.9)	29 (50)	0.45 **
Obesity (BMI > 30), *n* (%)	14 (24.1)	11 (22.9)	0.49 **
CHD, *n* (%)	7 (12)	6 (10.3)	0.77 **
Fever	31	31	1
Cough	23	17	0.24 **
Dyspnea	34	38	0.44 **
Headache	3	4	1
Asthenia	28	25	0.57 **
Dysgeusia	7	5	0.54 **
Anosmia	3	5	0.72 ***
Diarrhea	7	9	0.59 **
PaO2/FiO2 ratio, mean ± SD	268 ± 55.3	263.8 ± 51.6	0.33 *
Heparin, *n* (%)	58 (100)	58 (100)	1
Dexamethasone, *n* (%)	58 (100)	58 (100)	1
Remdesivir, *n* (%)	40 (68.9%)	38 (65.5%)	0.69 **
Vitamin D (ng/mL), mean ± SD	12.6 ± 6.5	14.5 ± 5.4	0.086 *
Severe deficiency (<10 ng/mL), *n* (%)	24 (41.4)	15 (25.9)	0.07 **
Mild to moderate (10–20 ng/mL), *n* (%)	20 (34.5)	30 (51.7)	0.06 **
Insufficiency (20–29.9 ng/mL), *n* (%)	14 (24.1)	13 (22.4)	0.82 **
PCR (mg/dL), mean ± SD	7.2 ± 6.2	9.12 ± 6.15	0.097 *
LDH (mU/mL), mean ± SD	325.8 ± 94.1	367.22 ± 108.64	0.03 *
D-dimer (ng/mL), median (Q1-Q3)	1.28 (0.6–3.2)	1.19 (0.6–3.2)	0.92 ****
Ferritin (ng/mL), median (Q1-Q3)	462.5 (292.15–1017.5)	575 (432.5–1014.5)	0.13 ****

SD: standard deviation; BMI: body mass index; * Students’ *t*-test, ** Chi-squared test, *** Fishers’ Exact Test, **** Mann–Whitney U test.

**Table 2:** Comparison between inflammatory markers at baseline (T0) and after one week (T1) among patients included in our study.

Variable	Group A			Group B		
	T0	T1	*p*-Value	T0	T1	*p*-Value
CRP, mean ± SD	7.2 ± 6.2	3.5 ± 3.2	<0.0001 *	9.1 ± 6.1	7.5 ± 6.4	0.0158
LDH, mean ± SD	325.8 ± 94.1	282.2 ± 107.2	0.006 *	367.2 ± 108.6	359.2 ± 108.9	0.6114 *
D-dimer, median (IQR)	1.28 (0.6–3)	0.7 (0.4–1.21)	<0.001 **	1.19 (0.6–3.2)	1.15 (0.52–4.43)	0.533 **
Ferritin, median (IQR)	462.5 (293–990)	520 (251–922)	0.0058 **	575 (434–986)	653 (360–1120)	0.54 **

* Paired students’ *t* test, ** paired Wilcoxon signed-rank test. Group A: vitamin D3 group, Group B: matched group. T0: admission; T1: 1 week evaluation; CRP: C-reactive protein; SD: standard deviation; LDH: lactate dehydrogenase; IQR: interquartile range.

**Table 3:** Logistic regression analyses on variables influencing mortality.

Variable	Univariate			Multivariate		
	OR	CI	*p*	OR	CI	*p*
Age (10 years)	1.48	0.95–1.31	0.081	1.03	0.51–2.08	0.935
Male gender	0.37	0.11–1.19	0.097	0.56	0.07–4.43	0.587
Comorbidities						
Liver diseases	1					
Diabetes	1.37	0.39–4.79	0.618			
COPD	7.76	1.79–33.6	0.006	72.3	3.57–1463	0.005
Hypertension	1.18	0.38–3.66	0.768			
CHD	2.51	0.60–10.52	0.209			
Neurological diseases	1.72	0.33–8.93	0.65			
Symptoms						
Fever	0.82	0.27–0.51	0.73			
Cough	1.16	0.37	3.6			
Headache	1					
Asthenia	0.28	0.07–1.08	0.064	0.26	0.04–1.58	0.153
Dyspnea	2.27	0.60–8.65	0.23			
Nausea	2.67	0.48–14.7	0.261			
Diarrhea	0.45	0.05–3.67	0.453			
Anosmia	1.04	0.12–9.18	0.969			
Dysgeusia	0.64	0.76–5.34	0.677			
Laboratory findings						
P/F	0.98	0.97–0.99	0.002	0.98	0.96–1.00	0.033
CRP	1.13	1.04–1.23	0.003	1.17	1.04–1.32	0.009
Ferritin	1.00	1.00–1.00	0.047	1.00	0.99–1.00	0.315
LDH	1.01	1.00–1.01	0.026	0.99	0.99–1.01	0.702
D-Dimer	0.99	0.82–1.19	0.912			
Albumin	0.95	0.63–1.43	0.797			
Vit. D baseline	1.07	0.97–1.18	0.152			
Treatment						
Heparin	1					
Vitamin D	0.233	0.06–0.88	0.032	0.06	0.06–0.58	0.015
Remdesivir	0.42	0.13–1.29	0.129	0.26	0.04–1.63	0.150
Corticosteroid	1					
CPAP/NIV	11.9	3.07–46.25	<0.001			
ICU	59.4	12.17–290	<0.001			

## Data Availability

Data are fully available in the manuscript.

## References

[1] Commission WMH (2019). Report of Clustering Pneumonia of Unknown Etiology in Wuhan City. http://wjw.wuhan.gov.cn/front/web/showDetail/2019123108989.

[2] New-Type Coronavirus Causes Pneumonia in Wuhan: Expert—Xinhua|English.news.cn. http://www.xinhuanet.com/english/2020-01/09/c_138690570.htm.

[3] World Health Organization (WHO) (2020). Coronavirus Disease (COVID-19), Report 191. https://www.who.int/docs/default-source/coronaviruse/situation-reports/20200729-covid-19-sitrep-191.pdf?sfvrsn=2c327e9e_2.

[4] De Vito A., Geremia N., Princic E., Fanelli C., Panu Napodano C.M., Muredda A.A., Fiore V., Maida I., Fois A.G., Babudieri S. (2021). Does Angiotensin II receptor blockers increase the risk of SARS-CoV-2 infection? A real-life experience. Eur. Rev. Med. Pharmacol. Sci..

[5] Yang J., Petitjean S.J.L., Koehler M., Zhang Q., Dumitru A.C., Chen W., Derclaye S., Vincent S.P., Soumillion P., Alsteens D. (2021). Molecular interaction and inhibition of SARS-CoV-2 binding to the ACE2 receptor. Nat. Commun..

[6] Ali A., Vijayan R. (2020). Dynamics of the ACE2-SARS-CoV-2/SARS-CoV spike protein interface reveal unique mechanisms. Sci. Rep..

[7] Vaira L.A., Hopkins C., Salzano G., Petrocelli M., Melis A., Cucurullo M., Ferrari M., Gagliardini L., Pipolo C., Deiana G. (2020). Olfactory and gustatory function impairment in COVID-19 patients: Italian objective multicenter-study. Head Neck.

[8] De Vito A., Geremia N., Fiore V., Princic E., Babudieri S., Madeddu G. (2020). Clinical features, laboratory findings and predictors of death in hospitalized patients with COVID-19 in Sardinia, Italy. Eur. Rev. Med. Pharmacol. Sci..

[9] Niu S., Tian S., Lou J., Kang X., Zhang L., Lian H., Zhang J. (2020). Clinical characteristics of older patients infected with COVID-19: A descriptive study. Arch. Gerontol. Geriatr..

[10] Zhou F., Yu T., Du R., Fan G., Liu Y., Liu Z., Xiang J., Wang Y., Song B., Gu X. (2020). Clinical course and risk factors for mortality of adult inpatients with COVID-19 in Wuhan, China: A retrospective cohort study. Lancet.

[11] Covino M., De Matteis G., Santoro M., Sabia L., Simeoni B., Candelli M., Ojetti V., Franceschi F. (2020). Clinical characteristics and prognostic factors in COVID-19 patients aged ≥80 years. Geriatr. Gerontol. Int..

[12] Fiore V., De Vito A., Fanelli C., Geremia N., Princic E., Nivoli A., Maida I., Lorettu L., Madeddu G., Babudieri S. (2021). Mood Reactive Disorders among COVID-19 Inpatients: Experience from a Monocentric Cohort. Med. Princ. Pract..

[13] Fiore V., Beretta R., De Vito A., Barac A., Maida I., Joeseph Kelvin D., Piu C., Lai V., Madeddu G., Rubino S. (2022). Emerging Clinical Features of COVID-19 Related Pancreatitis: Case Reports and Review of the Literature. Front. Med..

[14] Liu K., Chen Y., Lin R., Han K. (2020). Clinical features of COVID-19 in elderly patients: A comparison with young and middle-aged patients. J. Infect..

[15] Du R.H., Liang L.R., Yang C.Q., Wang W., Cao T.Z., Li M., Guo G.Y., Du J., Zheng C.L., Zhu Q. (2020). Predictors of mortality for patients with COVID-19 pneumonia caused by SARSCoV-2: A prospective cohort study. Eur. Respir. J..

[16] De Vito A., Fiore V., Princic E., Geremia N., Panu Napodano C.M., Muredda A.A., Maida I., Madeddu G., Babudieri S. (2021). Predictors of infection, symptoms development, and mortality in people with SARS-CoV-2 living in retirement nursing homes. PLoS ONE.

[17] Yu H.H., Qin C., Chen M., Wang W., Tian D.S. (2020). D-dimer level is associated with the severity of COVID-19. Thromb. Res..

[18] Zinellu A., De Vito A., Scano V., Paliogiannis P., Fiore V., Madeddu G., Maida I., Zinellu E., Mangoni A.A., Arru L.B. (2021). The PaO2/FiO2 ratio on admission is independently associated with prolonged hospitalization in COVID-19 patients. J. Infect. Dev. Ctries..

[19] Rodriguez-Guerra M., Jadhav P., Vittorio T.J. (2021). Current treatment in COVID-19 disease: A rapid review. Drugs Context.

[20] Saravolatz L.D., Depcinski S., Sharma M. (2022). Molnupiravir and Nirmatrelvir-Ritonavir: Oral COVID Antiviral Drugs. Clin. Infect. Dis..

[21] Xu J., Yang J., Chen J., Luo Q., Zhang Q., Zhang H. (2017). Vitamin D alleviates lipopolysaccharide-induced acute lung injury via regulation of the renin-angiotensin system. Mol. Med. Rep..

[22] Imai Y., Kuba K., Rao S., Huan Y., Guo F., Guan B., Yang P., Sarao R., Wada T., Leong-Poi H. (2005). Angiotensin-converting enzyme 2 protects from severe acute lung failure. Nature.

[23] COVID-19 Treatment Guidelines Panel Coronavirus Disease 2019 (COVID-19) Treatment Guidelines. National Institutes of Health. https://www.covid19treatmentguidelines.nih.gov/.

[24] Annweiler C., Hanotte B., Grandin de l’Eprevier C., Sabatier J.M., Lafaie L., Célarier T. (2020). Vitamin D and survival in COVID-19 patients: A quasi-experimental study. J. Steroid Biochem. Mol. Biol..

[25] Chiodini I., Gatti D., Soranna D., Merlotti D., Mingiano C., Fassio A., Adami G., Falchetti A., Eller-Vainicher C., Rossini M. (2021). Vitamin D Status and SARS-CoV-2 Infection and COVID-19 Clinical Outcomes. Front. Public Health.

[26] Dror A.A., Morozov N., Daoud A., Namir Y., Yakir O., Shachar Y., Lifshitz M., Segal E., Fisher L., Mizrachi M. (2022). Pre-infection 25-hydroxyvitamin D3 levels and association with severity of COVID-19 illness. PLoS ONE.

[27] Liu P.T., Stenger S., Li H., Wenzel L., Tan B.H., Krutzik S.R., Ochoa M.T., Schauber J., Wu K., Meinken C. (2006). Toll-like receptor triggering of a vitamin D-mediated human antimicrobial response. Science.

[28] Wang T.T., Nestel F.P., Bourdeau V., Nagai Y., Wang Q., Liao J., Tavera-Mendoza L., Lin R., Hanrahan J.W., Mader S. (2004). Cutting edge: 1,25-dihydroxyvitamin D3 is a direct inducer of antimicrobial peptide gene expression. J. Immunol..

[29] Kim J., Yang Y.L., Jang S.H., Jang Y.S. (2018). Human beta-defensin 2 plays a regulatory role in innate antiviral immunity and is capable of potentiating the induction of antigen-specific immunity. Virol. J..

[30] Barlow P.G., Svoboda P., Mackellar A., Nash A.A., York I.A., Pohl J., Davidson D.J., Donis R.O. (2011). Antiviral activity and increased host defense against influenza infection elicited by the human cathelicidin LL-37. PLoS ONE.

[31] Ul Afshan F., Nissar B., Chowdri N.A., Ganai B.A. (2021). Relevance of vitamin D3 in COVID-19 infection. Gene Rep..

[32] Martha J.W., Wibowo A., Pranata R. (2021). Prognostic value of elevated lactate dehydrogenase in patients with COVID-19: A systematic review and meta-analysis. Postgrad. Med. J..

[33] Martineau A.R., Jolliffe D.A., Hooper R.L., Greenberg L., Aloia J.F., Bergman P., Dubnov-Raz G., Esposito S., Ganmaa D., Ginde A.A. (2017). Vitamin D supplementation to prevent acute respiratory tract infections: Systematic review and meta-analysis of individual participant data. BMJ.

[34] Bouillon R., Marcocci C., Carmeliet G., White J.H., Dawson-Hughes B., Lips P., Munns C.F., Lazaretti-Castro M., Giustina A., Bilezikian J. (2019). Skeletal and extraskeletal actions of Vitamin D: Current evidence and outstanding questions. Endocr. Rev..

[35] Quesada-Gomez J.M., Entrenas-Castillo M., Bouillon R. (2020). Vitamin D receptor stimulation to reduce acute respiratory distress syndrome (ARDS) in patients with coronavirus SARS-CoV-2 infections. J. Steroid Biochem. Mol. Biol..

[36] Annweiler G., Corvaisier M., Gautier J., Dubée V., Legrand E., Sacco G., Annweiler C. (2020). Vitamin D supplementation associated to better survival in hospitalized frail elderly COVID-19 patients: The GERIA-COVID quasi-experimental study. Nutrients.

[37] Entrenas Castillo M., Entrenas Costa L.M., Vaquero Barrios J.M., Alcalá Díaz J.F., López Miranda J., Bouillon R., Quesada Gomez J.M. (2020). Effect of calcifediol treatment and best available therapy versus best available therapy on intensive care unit admission and mortality among patients hospitalized for COVID-19: A pilot randomized clinical study. J. Steroid Biochem. Mol. Biol..

[38] Murai I.H., Fernandes A.L., Sales L.P., Pinto A.J., Goessler K.F., Duran C.S.C., Silva C.B.R., Franco A.S., Macedo M.B., Dalmolin H.H.H. (2021). Effect of a single high dose of Vitamin D3 on hospital length of stay in patients with moderate to severe COVID-19: A randomized clinical trial. JAMA.

[39] Jaun F., Boesing M., Lüthi-Corridori G., Abig K., Makhdoomi A., Bloch N., Lins C., Raess A., Grillmayr V., Haas P. (2022). High-dose vitamin D substitution in patients with COVID-19: Study protocol for a randomized, double-blind, placebo-controlled, multi-center study-VitCov Trial. Trials.

